# Constraints on Acceleration in Bilingual Development: Evidence from Word Segmentation by Spanish Learning Infants

**DOI:** 10.3390/bs14020108

**Published:** 2024-02-01

**Authors:** Victoria Mateu, Megha Sundara

**Affiliations:** 1 Department of Spanish and Portuguese, University of California, Los Angeles, CA 90095, USA; 2 Department of Linguistics, University of California, Los Angeles, CA 90095, USA

**Keywords:** Spanish, English, bilingualism, word segmentation, acceleration, frequency, noise tolerance, regularization

## Abstract

We have previously shown that bilingual Spanish and English-learning infants can segment English iambs, two-syllable words with final stress (e.g., guiTAR), earlier than their monolingual peers. This is consistent with accelerated development in bilinguals and was attributed to bilingual infants’ increased exposure to iambs through Spanish; about 10% of English content words start with an unstressed syllable, compared to 40% in Spanish. Here, we evaluated whether increased exposure to a stress pattern alone is sufficient to account for acceleration in bilingual infants. In English, 90% of content words start with a stressed syllable (e.g., KINGdom), compared to 60% in Spanish. However, we found no evidence for accelerated segmentation of Spanish trochees by Spanish-English bilingual infants compared to their monolingual Spanish-learning peers. Based on this finding, we argue that merely increased exposure to a linguistic feature in one language does not result in accelerated development in the other. Instead, only the acquisition of infrequent patterns in one language may be accelerated due to the additive effects of the other language.

## 1. Introduction

Language development depends, at least in part, on lexical knowledge. One of the first challenges that infants face in this domain is the segmentation of words from fluent, continuous speech. A number of experimental studies have found that infants use prosodic cues to segment words. For instance, because 90% of English content words begin with a stressed syllable, e.g., KINGdom [1], monolingual English-learning infants learn to associate stress with word onsets (Metrical Segmentation Strategy [2]). As a result, by 8 months, they can segment trochees from continuous speech, but they fail to segment iambs, e.g., guiTAR [3,4]. It is not until 10.5 months of age that monolingual English-learning infants segment iambs as well [4]. Converging evidence that infants associate stressed syllables with word onsets comes from infants learning other languages with predominant trochaic patterns, such as German [5] and Dutch [6].

In a recent study [7], we have shown that bilingual infants learning Spanish and English successfully segment English iambs at an earlier age than their monolingual English peers. As in Jusczyk et al. [4], we used the Head-turn Preference Procedure to familiarize bilingual 8-month-old infants with two iambic words (e.g., ‘beret’ and ‘guitar’ or ‘device’ and ‘surprise’) embedded in passages until they accumulated 45 s of listening time to each. In the test phase, infants heard four different word lists, two with familiar words and two with novel words. Results showed that bilingual Spanish-English 8-month-olds listened significantly longer to the familiar words compared to the novel words. Thus, bilingual infants successfully segmented iambs from continuous speech two months before their monolingual English-learning peers. That is, bilingual infants showed accelerated development, as defined by Paradis and Genesee [8]—the earlier acquisition of a linguistic property in bilinguals compared to monolinguals.

In the linguistic domain, accelerated development in bilinguals’ speech production and perception has been reported in only a handful of studies, and therefore the conditions that lead to accelerated linguistic development are still a matter of debate. The purpose of this study is to evaluate various proposals to determine the conditions under which bilinguals show accelerated development. Below, we discuss findings on accelerated development in bilinguals and four non-mutually exclusive hypotheses proposed to account for it: (i) language dominance accounts; (ii) perceptual salience accounts; (iii) domain-general accounts; and (iv) frequency accounts. We evaluate the hypotheses against published findings about Spanish-English bilingual infants accelerated segmentation of Spanish iambs [7]. Then, we derive predictions for whether Spanish-English bilingual infants’ ability to segment Spanish trochees should be accelerated. In Section 2 and Section 3, we present data from two new experiments testing Spanish-English bilingual 8-month-olds’ segmentation of Spanish trochees. In anticipation of our results, our findings are not consistent with any of the hypotheses that have been proposed to date. In the Discussion, we present a novel account for accelerated development in bilingual infants, arguing for a more restricted role of frequency.

### 1.1. Previous Proposals to Account for Acceleration

#### 1.1.1. Language Dominance Accounts

A popular proposal when bilingual development diverges from that of monolinguals is that it is due to cross-linguistic transfer [9,10,11,12,13,14,15,16]. Acceleration, then, is a form of positive transfer from the dominant language to the non-dominant language.

Despite being a popular construct, there is no consensus on how language dominance should be indexed. In some studies dominance is defined at the individual level, based on the relative amount of input a specific child receives in each language. In others, it is defined at the community level, based on which language is used most often outside the home environment. In some studies, dominance is treated as a binary variable (i.e., dominant or non-dominant); in others, it is treated as a continuous variable indexed by the percent input in one language. Regardless of the way it is measured, dominance does not consistently predict acceleration. 

For instance, Lleó and colleagues [17,18] propose that the accelerated rate of development in the production of Spanish codas (e.g., “azul” blue [aθul]) in Spanish-German bilingual children (1;1–2;3) living in Germany, when compared to age-matched Spanish monolinguals, is due to language dominance. German has more codas (63%) compared to Spanish (28%; [19]; more on frequency effects below). They argue that the language of the community, in this case German, is dominant and thus plays a role in determining the direction of positive transfer between the two languages of a bilingual child. Further evidence for this proposal comes from Stahnke’s [20] data on the acquisition of French determiners in two French-Italian bilingual children—one French-dominant child who lived in Paris and one Italian-dominant child who lived in Rome. The bilingual child living in Rome outperformed the bilingual child living in Paris on the production of French determiners, possibly due to positive transfer from the child’s community language, Italian, which has more prosodically prominent determiners than French (more on prosodic salience effects below).

However, there are many studies that have failed to confirm the role of dominance in predicting acceleration in developing bilinguals. Italian monolingual children reach adult-like levels of determiner use earlier than their German counterparts; thus, positive transfer is expected from Italian to German when Italian is the dominant language at the individual or community level, but not when German is dominant or when the child receives balanced input, e.g., [21]. Kupisch [21] examined the production of determiners by four Italian-German bilingual children living in Germany. The Italian-dominant bilingual child supplied German determiners in obligatory contexts more frequently than their monolingual German counterparts, despite living in Germany. This acceleration is not predicted if dominance is evaluated at the level of the community. Additionally, the two balanced bilingual children who received comparable input in the two languages also showed evidence of acceleration in German. This is inconsistent with a role for dominance as an individual level construct because acceleration was observed even when there was no difference in relative input in the two languages. Similarly, and counter to the predictions of dominance-based accounts, Kupisch [22] found that two French-German children showed an accelerated rate of development in the production of German determiners compared to monolingual German children, regardless of relative differences in the amount of input they received in each language. 

In experiments evaluating word segmentation as well, there is no evidence that language dominance, whether indexed at the community level or at the individual level, predict acceleration. The bilingual Spanish-English learning infants tested by Mateu and Sundara [7] were living in Los Angeles, where English is, arguably, the community language, and nevertheless, their segmentation of English iambs was accelerated due to experience with Spanish, inconsistent with a prediction based on community standard of dominance (see also [23] for absence of dominance effects on word segmentation by French-English bilingual infants). Mateu and Sundara also failed to find any relationship between the percentage of Spanish input and the segmentation of iambs in English. So, their findings on word segmentation do not support a role for dominance determined at the individual level in predicting acceleration either.

#### 1.1.2. Perceptual Salience Accounts

It has also been proposed that acceleration is observed only in cases where bilinguals are acquiring linguistic features that are perceptually more salient and thus more easily learned in one language than the other. As mentioned above, both Italian-German bilingual children and French-German bilingual children outpace their monolingual German peers in their production of German determiners [21,22]. Kupisch attributes this to several properties of Italian (and French) determiners, which make them more perceptually salient and thus easier to acquire than German determiners. Italian and French determiners are proclitic on the noun, while in German they often form a prosodic unit with the previous word and are therefore less prosodically prominent. Moreover, the vocalic endings of most Italian DPs harmonize with the vowel in the article, e.g., *una/la ragazza* “a/the girl”, while in German they do not. Kupisch argues that Italian-German and French-German bilingual children benefit from the perceptual salience of determiners in Italian (and French), and as a result of positive transfer, they show an accelerated rate of acquisition of determiners in German compared to their monolingual German peers.

Similarly, Stahnke [20] also finds supporting evidence for the role of perceptual salience in the accelerated production of determiners. French articles are all monosyllabic, whereas in Italian a few are disyllabic, which makes the latter more prosodically prominent. Moreover, Italian has considerably more multisyllabic nouns than French, which, according to Lleó and Demuth [24], allows for an earlier prosodification of determiners in Italian than in French. Based on an analysis of a longitudinal corpus with three children, Stahnke shows that one Italian-dominant bilingual child outperformed a monolingual French learning child, consistent with acceleration due to the more salient property of determiners in Italian.

The results from Mateu and Sundara [7] on word segmentation, however, cannot be accounted for by appealing to perceptual salience. Spanish iambs cannot be described as more prosodically salient than English iambs. In both languages, stress is lexical and is instantiated by lengthening. In fact, stress is arguably more salient in English than in Spanish, since there is a greater difference in duration between stressed and unstressed syllables in English than in Spanish [19,25], and stressed and unstressed syllables in English additionally differ in vowel quality—unstressed syllables tend to have reduced vowels [26,27,28,29]. Therefore, perceptual salience accounts fail to predict the positive transfer of stress-based segmentation strategies from Spanish to English.

#### 1.1.3. Domain-General Accounts

Acceleration has also been proposed to result from the mere fact that the individual is bilingual. There are many studies showing that bilingualism enhances general (and language-specific) cognitive performance, i.e., executive functioning, e.g., [30,31,32,33,34,35,36,37]. This has been attributed to bilinguals’ experience with language inhibition—while speaking one language, bilingual speakers must concurrently suppress the other language, thus exercising executive control. In turn, many researchers have argued that enhanced executive control can result in positive linguistic outcomes [38,39,40,41,42,43,44,45,46]. Overall, though, if bilinguals have an across-the-board enhancement of cognitive performance that is at the root of acceleration, it is difficult to reconcile why acceleration is so seldom documented in the literature.

Instead, Müller [47] argues that sometimes bilinguals rely on domain-general mechanisms in response to superficial similarities in their two languages, and this is mischaracterized as acceleration (see also [48,49]). For example, bilingual learners of German in combination with a Romance language, such as Italian or French, tend to correctly place finite verbs in second position in German [50]. This is in contrast to age-matched monolingual German children who erroneously place finite verbs in the final position [51]. On the surface, this looks like acceleration. However, Romance languages typically follow a Subject-Verb-Object order, so verbs often appear in second position. In German, the placement of the verb is variable—finite verbs move to second position in matrix clauses but remain in situ, in final position in embedded clauses. Müller contends that bilingual children succeed earlier simply by drawing on surface-level word order parallels in their two languages. Consistent with this account, although bilingual children correctly place finite verbs in second position in German earlier than their monolingual peers, approximately half of the bilingual children who skip the non-target-like verb-final stage face challenges with verb placement in German embedded clauses [51]. 

Two similar proposals (NLM-e model [52] and the Perceptual Wedge Hypothesis [53]) have also been posited to account for bilingual infants’ putatively advanced abilities in speech perception. Specifically, bilingual infants continue to show neural responses to non-native speech sound distinctions when their monolingual peers have stopped responding to them. The bilinguals’ responses in these cases have been shown to reflect their reliance on auditory perceptual sensitivities rather than specific language experience as demonstrated by their monolingual peers [53,54,55,56], which suggests that bilingual experience may promote extended flexibility or openness to learning native and non-native contrasts because of their extended reliance on auditory perceptual sensitivities.

Mateu and Sundara [7], however, present evidence against the proposal that the earlier segmentation of English iambs by bilingual Spanish-English infants is due to their reliance on domain-general mechanisms, specifically their ability to leverage transitional probabilities to identify word boundaries. Bilingual adults [57,58] as well as infants [59,60,61,62] have been demonstrated to have an advantage when using transitional probabilities to find words in artificial languages. However, Mateu and Sundara show that bilingual infants failed to segment Spanish iambs, given comparable statistical cues. Thus, there is no evidence that bilingual 8-month-olds rely solely on transitional probabilities to segment words in their native languages or that this is the reason for their earlier segmentation of English iambs.

#### 1.1.4. Frequency Effects

Often, a necessary condition in proposals about acceleration is more evidence for a linguistic feature in one language compared to the other. This hypothesis is sometimes referred to as additiveness [63] and is supported by the widest range of studies: those investigating phonological and morphosyntactic production in bilingual children as well as those based on word segmentation by bilingual infants. 

Recall that Lleó et al. [17] found that bilingual Spanish-German children produce codas in Spanish before their monolingual counterparts. As mentioned previously, codas are more frequent in German than in Spanish, and therefore, bilingual Spanish-German children hear and, consequently, produce more codas than their monolingual Spanish counterparts. Similarly, Tamburelli, Sanoudaki, Jones, and Sowinska [64] found acceleration in the acquisition of complex consonant clusters in Polish-English bilinguals—Polish-English bilinguals outperform English monolinguals (7; 0–8; 11) when producing/s/+ obstruent clusters in English non-words. Thus, exposure to Polish, a language with more complex onsets, facilitates the acquisition of English, a language with fewer complex onsets.

A frequency account could also explain some of the results on the production of determiners. Kupisch [65] found that overall, the number of bare nouns, i.e., nouns that are not preceded by a determiner, is highest in German (18%), lower in Italian (12%), and lowest in French (6%). Possibly as a result of exposure to languages where production of determiners is obligatory more often, Italian-German and French-German bilingual children produce more determiners in German compared to monolingual German children [21,22]. A frequency-based account, however, cannot explain why two Italian-French bilingual children showed positive transfer from Italian to French [20], indicating that differences in frequency alone are inadequate to capture all cases of acceleration.

Additiveness effects have been found in the domain of morphosyntactic acquisition as well. Specifically, it has been reported that English wh-questions are acquired earlier in Spanish-English bilinguals than in English monolinguals [66]. Hsin [66] claims that the facilitating effect of Spanish on English wh-question production stems at least in part from the frequent exposure to and early mastery of other operations utilizing the C-domain in Spanish. For example, whereas object topicalization, a construction that involves movement to CP, appears in 1.35% of all finite clauses in Spanish [67], it only appears in 0.00134% of English finite clauses [68,69], i.e., topicalizations are 1000 times more frequent in Spanish than in English. 

Lastly, Mateu and Sundara [7] also attribute the acceleration effects found in segmentation of English iambs to the effects of additiveness. In Spanish, stress placement in words is more variable than in English. Approximately 40% of prosodic words in Spanish child-directed speech start with a weak syllable, compared to only 10% in English [70]. Therefore, Spanish-English bilingual infants hear more iambs compared to their monolingual English peers because of their input in Spanish. The authors argue that because of their greater experience with iambs, Spanish-English bilingual infants are able to associate stressed syllables with offsets at an earlier developmental point than their monolingual peers.

### 1.2. The Current Study

In two experiments, we evaluated whether 8-month-old bilingual Spanish-English-learning infants segment Spanish trochees earlier than their monolingual peers. Thus far, the word segmentation abilities of Spanish-learning infants have been examined in two studies. Based on these studies, we know that monolingual Spanish infants can segment monosyllabic words embedded in passages by 6 months [71]. However, we do not know when monolingual Spanish-learning infants segment disyllabic words. In Mateu and Sundara [7], we tested Spanish monolingual and Spanish-English bilingual infants’ ability to segment Spanish iambs; neither group succeeded. Therefore, if at all Spanish monolingual 8-month-olds are able to segment disyllabic words, it remains unclear. In both experiments, we present data on monolingual Spanish-learning 8-month-olds as well to address this question. Crucially, if bilingual infants succeed at segmenting Spanish trochees when Spanish monolingual infants fail, the result will provide evidence of accelerated bilingual development. 

All four hypotheses discussed above predict accelerated segmentation of Spanish trochees by Spanish-English bilingual infants. We tested all infants in Los Angeles, where English, arguably, is the community language. We know from previous research that English-learning infants segment English trochees by 8 months [4]. So based on their English experience, Spanish-English-learning bilingual 8-month-olds should be able to segment Spanish trochees as well. Thus, based on a community construct of dominance, we expected accelerated segmentation of Spanish trochees. We also evaluated the dominance account at the individual level, where bilingual infants’ acceleration is expected to be commensurate with the percent of English input. In a salience-based account as well, bilingual infants’ segmentation of Spanish trochees is predicted to be accelerated because, as mentioned above, stress cues are more perceptually salient in English than in Spanish. Similarly, we expected to see acceleration based on the enhanced executive functioning hypothesis as well; bilingual infants are better able to track transitional probabilities than their monolingual peers. Finally, in English, 90% of content words start with a stressed syllable [1], compared to 60% in Spanish [72]. As a result of more exposure to trochees in English, following the additiveness account as well, we expected to see acceleration in Spanish-English bilingual infants.

## 2. Experiment 1

In Experiment 1, we familiarized Spanish-English bilingual 8-month-olds and their monolingual Spanish peers with Spanish trochees embedded in passages, then tested them on the Spanish trochees produced in isolation. Because we wanted to maximize the chances of success, all target words were of the form CVC.CV, since in Spanish, 95% of words of this syllable shape are trochees [72], and Spanish-learning infants have been shown to be sensitive to syllable weight when detecting stress at around 9 months [72]. Additionally, we have previously shown that monolingual English-learning 8-month-olds are able to segment these Spanish trochees when given an extended familiarization phase of 60 s [73]. Thus, we know that these Spanish trochees can be segmented from passages by infants at 8 months.

### 2.1. Materials and Methods

#### 2.1.1. Participants

We based our sample size on a meta-analysis of 285 experiments from 68 publications on infant word segmentation abilities [74]. The mean age of infants tested in the meta-analysis was 8 months (range = 6–25 months). The median sample size was 20 (range = 4–64 participants), with an aggregate effect size (Cohen’s *d*) of 0.16 (*SE* = 0.03). We therefore aimed for at least 20 participants in each group. 

The final sample included data from 29 Spanish-English bilingual 8-month-olds (*M* = 8.49, range = 7.5–9.7). Only infants who were exposed to Spanish between 20% and 80% of the time were included, so we could compare their performance to the group of bilingual infants tested on English iambs in Mateu and Sundara [7]. Based on detailed language questionnaires, we calculated the number of hours per week each infant heard the two languages [75]. Exposure to Spanish ranged from 22.7 to 76.6% (M = 47.5%). Based on the parental report, none of the participants had a history of cognitive impairment or an ear infection on the day of testing. Four more bilingual infants were tested but excluded because they failed to complete the task due to fussiness. Additionally, 30 monolingual Spanish-learning 8-month-olds (*M* = 8.38, range = 7.5–9.5) who had at least 80% of exposure to Spanish (*M* = 95.1%, range = 80.9–100%) were included in the study as a control group. Thus, the language profile of monolingual Spanish infants in this experiment was also similar to the group of monolingual infants in Mateu and Sundara [7]. Four more monolingual infants were tested but excluded due to fussiness. All infants were recruited from Los Angeles county, and none of them were tested in the experiments reported in [7].

#### 2.1.2. Stimuli

The same Spanish stimuli used in Sundara and Mateu [73] were employed in this study. The four CVC.CV trochaic words were “gancho” *hook* [‘gan.tʃo], “salsa” *sauce* [‘sal.sa], “gesto” *gesture* [‘hes.to], and “venda” *bandage* [‘ben.da]. Each passage had six sentences, with the target word occurring once per sentence, twice at the beginning, twice in the middle, and twice at the end (see Appendix A). The lists consisted of repetitions of the target words in isolation. Both passages and lists were recorded by a female native speaker of Mexican Spanish who was asked to read the sentences and words in infant-directed speech. 

The acoustic properties of the stimuli are described in detail in Sundara and Mateu [73] and summarized here. As in the case of the English and Spanish iambic stimuli in Mateu and Sundara [7], duration was the only reliable cue to distinguish the first and second syllable in the passages and the lists of the Spanish trochees; the first stressed syllable (passages, 300 ms; lists, 343 ms) was significantly longer than the second, unstressed one (passages, 220 ms; lists, 267 ms). Pitch and intensity were not reliably different in either passages or lists. As reported in [73], the target trochaic words had a backward transitional probability of 0.33, meaning they were preceded by one of three different syllables, and a mean forward transitional probability of 0.18, meaning they were followed by one of five or six different syllables. The English iambs employed in Mateu and Sundara [7] had comparable transitional probabilities, with slightly strong (i.e., lower) backward TP (0.17) and slightly weaker (i.e., higher) forward TP (0.23). 

#### 2.1.3. Procedure and Design

The Headturn Preference Procedure (HPP), as described by Jusczyk, Houston, and Newsome [4], was used to test segmentation of Spanish trochees from fluent speech. Infants sat on their caregiver’s lap in the center of a three-sided booth with three lights at the center of each panel. The two side panels also had a loudspeaker mounted behind the light. At the beginning of each trial, the light on the center panel flashed, attracting the infant’s gaze. When the infant was oriented to the center panel’s flashing light, the center light stopped flashing, and one of the lights on the side panels began to flash. When the infant turned their head and looked towards the side light, an auditory stimulus started to play. The audio presentation continued until the infant looked away from the flashing light for more than two consecutive seconds or when the trial ended (max duration = 17 s). 

The experiment consisted of two phases. In the familiarization phase, half the infants heard two passages with either *gancho* and *salsa* or *gesto* and *venda.* Once an infant accumulated 45 s of listening time to each passage, the test phase started. In the test phase, infants were presented with four separate word lists (two familiar and two novel) in three blocks (12 trials total). The experimenter was seated outside the booth and observed the live feed on a computer screen connected to the video camera. She recorded the direction and duration of the infant’s head turns, which determined the stimulus presentation. Both the caregiver and experimenter wore noise-canceling headphones and listened to music with lyrics to eliminate potential biases. Testing lasted approximately 5–7 min.

### 2.2. Analyses

Listening times to the familiar and novel trials in the test phase were modeled with Bayesian linear mixed effects in the *R* programming environment [76] using the package *brms* [77]. In Bayesian statistics, the goal of modeling is to estimate parameters (i.e., *β* coefficients) that define the relationship between variables of interest—in this case, the relationship between Group (also Condition, Block), and trial type—on the dependent variable, Listening Time. The outcome is a probability distribution that indicates the plausibility of different values of the parameters of interest. A strength of this approach is that it can quantify support for the null hypothesis, unlike in frequentist analyses. Moreover, Bayesian methods do not require large sample sizes to produce accurate results, as they do not depend on the asymptotic properties of estimators. This can be especially useful in infant studies, which often rely on small sample sizes due to the considerable time, cost, and effort required to collect data from infants. 

The model was estimated using NUTS sampling with 4 chains of 8000 iterations and a warmup of 1000. For the population-level effects, we set a default normal prior with a mean of 0 and a standard deviation of 1. We chose a weakly informative prior because we believed the coefficients to be small at best. For the intercept of the model, we set a prior with a weakly informative mean of 0 and a standard deviation equal to the standard deviation of infants’ listening time in milliseconds (SD = 4634), allowing the intercept to vary widely. For the residual standard deviation σ of the model, we chose a mean of 1 and a standard deviation of 0.5. For the full analysis code, see OSF page.

Fixed effects included Group (Bilingual, Monolingual) and Condition (gancho.salsa, gesto.venda) as between-subjects variables and Block (1, 2, 3) and Trial Type (familiar, novel) as within-subjects variables, and all interactions. We also included random intercepts for subjects to model baseline differences in listening time (full model: ListeningTime ~ Group * Block * Condition * TrialType + (1|subject)). Because percent of English input did not contribute to the model fit when examining the bilingual data, we do not report analysis with percent English as a variable. Planned comparisons were performed with the *emmeans* package [78]. 

Below, we report the median estimated coefficient of each variable of interest (*β*) and its 95% Credible Interval (CI). The narrower the CI, the more precise the estimate of the effect. The effect is considered credible when the CI does not include 0. When the CI includes 0, we report the probability of direction (pd); the pd varies between 50 and 100 and summarizes the percentage of estimated coefficients that have the same direction as the median coefficient *β*. If the pd is around 50, we can be confident that the independent variable does not contribute to model fit. Our estimations for all population-level effects converged (Rhats = 1.00 for all parameters).

### 2.3. Results

**Figure 1:**
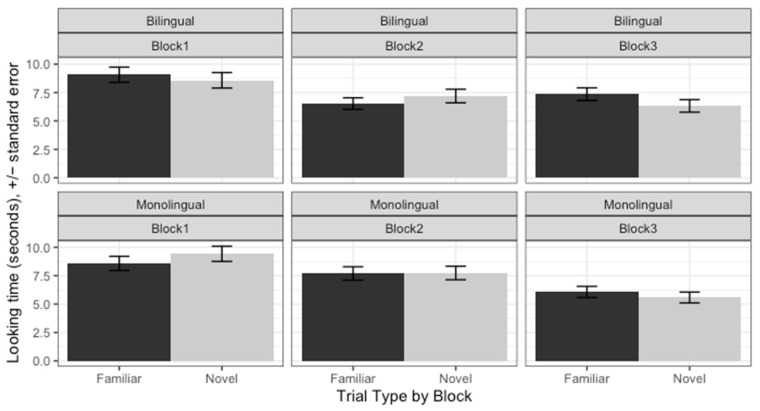
Listening times broken down by Block from Experiment 1 where we tested segmentation of Spanish trochees by bilingual Spanish-English-learning infants (**upper panel**) and monolingual Spanish-learning infants (**lower panel**).

Overall, the Bayesian analysis showed that both Spanish-English bilingual infants and their Spanish monolingual peers segmented Spanish trochees in Block 3; that is, we found no evidence for acceleration. Listening time data in each of the three blocks for bilingual Spanish-English and monolingual Spanish infants is presented in Figure 1. There was a credible three-way interaction between Trial Type, our variable of interest, and Block and Group (*β* = 2.13, [0.16, 4.09]). Post hoc comparisons revealed that both bilingual (*β* = 4.398, [1.24, 7.49]) and monolingual infants (*β* = 6.927, [2.63, 11.28]) listened credibly longer at familiar words compared to novel words in Block 3. Listening times in no other blocks were credibly different. The interaction stemmed from a larger estimate in the monolingual group. Crucially, there was no main effect of Group (*β* = 0.00, [−1.96, 1.97], pd = 50.00%) nor an interaction of Trial Type by Group (*β* = 0.21, [−1.75, 2.17], pd = 58.39%). Because both estimates are centered around 0 with a pd less than 60%, we can be confident that there were no consistent differences between the Groups. The Full model results are reported on the project OSF page. 

## 3. Experiment 2

Experiment 2 was designed to confirm that both monolingual and bilingual infants were segmenting the complete trochaic target word and not only the stressed syllable (based on Jusczyk, Houston, and Newsome’s [4] argument applied to segmentation of English iambs). In this experiment, we familiarized infants with the same passages containing the Spanish trochees, but in the test phase, they were presented with lists of the stressed syllables alone. If monolingual or bilingual infants in Experiment 1 were only segmenting the stressed syllable and not the entire trochee, we expected them to listen longer to the stressed syllable alone in this experiment. If monolingual infants only segment the stressed syllable, whereas bilinguals segment the whole disyllabic word, this would also be evidence for acceleration. The opposite pattern, where bilingual infants segment only the stressed syllable whereas monolinguals segment the whole disyllabic word, would be evidence of a delay.

### 3.1. Materials and Methods

#### Participants

All inclusion criteria were identical to those in Experiment 1. In the final sample, data from 24 bilingual Spanish-English-learning 8-month-olds (*M* = 8.37, range = 7.6–9.5) who had an average exposure to Spanish of 44.3% (range = 20% to 73.1%) were included. Eight more bilingual infants were tested, but their data was excluded because they did not complete the testing due to fussiness. Additionally, 28 monolingual Spanish-learning 8-month-olds (*M* = 8.6, range = 7.5–9.5) who had more than 80% exposure to Spanish (*M* = 94.8%, range = 81–100%) were included in the study as the control group. One more monolingual infant was tested but excluded due to fussiness. None of the infants participated in Experiment 1 or in the experiments reported in [7].

### 3.2. Stimuli, Procedure and Design, and Analysis

The stimuli, procedure, design, and analysis (see OSF page) were the same as in Experiment 1, except that infants were presented with just the stressed syllable of the trochaic word—*gan*, *sal*, *ges*, and *ven*—in the test phase. Again, percent of English exposure was not a significant predictor in the model, so we do not report analysis with percent of English as a variable. 

### 3.3. Results and Discussion

**Figure 2:**
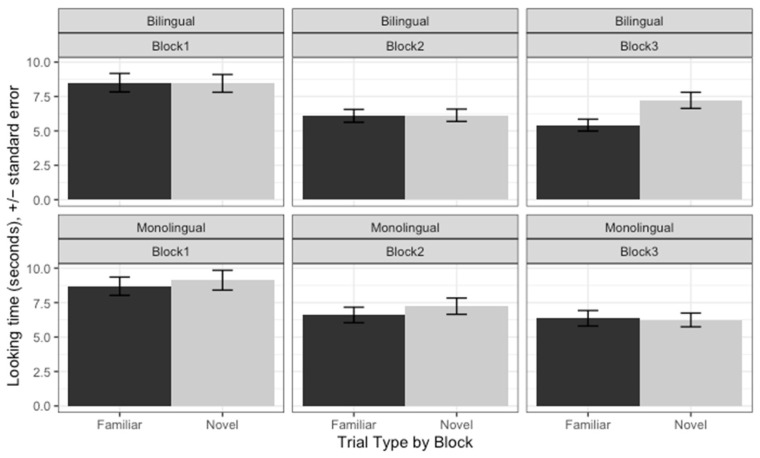
Listening times broken down by Block from Experiment 2 where we tested the segmentation of the stressed syllable of Spanish trochees by bilingual Spanish-English-learning infants (**upper panel**) and monolingual Spanish-learning infants (**lower panel**).

Again, using Bayesian analysis, we found no evidence that either bilingual Spanish-English-learning infants or their monolingual Spanish peers were segmenting the stressed syllable alone. Listening time data in each of the three blocks for both groups is presented in Figure 2. Neither the effect of Trial Type nor any interactions with Trial Type were credible. Note that although the listening times of bilingual infants in Block 3 for novel words are numerically greater than those for familiar words, the difference is not statistically reliable, as shown by a CI centered close to 0 (*β* = 0.268, [−2.81, 3.32]). Full model results are reported on the project OSF page. In the absence of a difference between the bilingual and monolingual groups, we have no evidence for acceleration—or delay—in bilingual Spanish-English-acquiring infants. That is, in Experiment 2 as well, bilingual Spanish-English 8-month-olds behaved like monolingual Spanish-learning 8-month-olds.

## 4. General Discussion

In this study, we sought to identify the conditions under which bilingual development is accelerated. In Experiment 1, we tested bilingual Spanish-English 8-month-olds and their monolingual Spanish peers on their ability to segment Spanish trochees. Our results showed an identical pattern of results for monolingual and bilingual infants—longer listening times to the familiar words in the third block. Further, the performance of bilingual infants was not correlated with the extent of English input. Results from Experiment 2 further confirmed that neither group was simply extracting the stressed syllable out of the trochee, providing further evidence that there was no difference in the performance of bilingual Spanish-English and monolingual Spanish infants. Thus, infants in both groups behaved similarly—they did not segment the stressed syllable in Experiment 2, but successfully segmented the whole trochee in Block 3 in Experiment 1. We first discuss the evidence for successful segmentation of Spanish trochees by Spanish-learning bilinguals and monolinguals, then revisit the four hypotheses predicting acceleration in bilingual infants. 

Recall that although there is evidence that monolingual Spanish-learning infants can segment Spanish monosyllabic words [71], it is unclear whether they can segment disyllabic words. Mateu and Sundara [7] did not find evidence that Spanish-learning 8-month-olds, whether monolingual or bilinguals, segment Spanish iambs. We present the first evidence that Spanish-learning 8-month-olds are able to segment Spanish trochees. Monolingual infants listened credibly longer to previously familiarized Spanish trochees in Experiment 1 and not to the stressed syllable alone in Experiment 2. Note, however, that we saw evidence for credible segmentation of Spanish trochees in Block 3 alone (Experiment 1). We are now testing monolingual Spanish-learning infants to delineate the developmental trajectory of word segmentation in Spanish.

Crucially for our research question, Spanish-English bilingual infants as well, just like the monolingual Spanish learning infants, credibly segmented Spanish trochees in Block 3. Therefore, contrary to the predictions of all four hypotheses, we saw no evidence of accelerated segmentation of Spanish trochees in bilingual infants. Our results are clearly incompatible with language dominance accounts. In dominance-based accounts, bilinguals transfer knowledge from their dominant language, which can lead to accelerated development in their non-dominant language (e.g., [14,18]). We included bilingual infants with exposure to English ranging from 20% to 80%, and yet bilingual infants’ success at segmenting Spanish trochees was not predicted by percent of exposure to English. Further, recall that all Spanish-learning infants were tested in Los Angeles, where the language of the community is English. So dominance, whether defined using a continuous measure at the individual level or in a categorical way based on the community language, did not predict acceleration.

The findings presented here are also inconsistent with proposals where the acquisition of more perceptually prominent linguistic elements results in accelerated sensitivity to the less prominent feature. Stress is arguably more salient in English than in Spanish. Thus, exposure to English stress should have accelerated Spanish learning infants’ segmentation of Spanish trochees, but that was not the case. Mateu and Sundara’s previous finding that Spanish-English-learning infants’ ability to segment English iambs is accelerated is also inconsistent with a perceptual salience-based account; stress is not as salient in Spanish as in English, and nevertheless, it is exposure to Spanish that accelerated segmentation of English iambs.

Proposals that claim that acceleration is due to bilingual infants relying on a more efficient, non-linguistic computation also fail to account for either the findings reported here or the ones previously reported in Mateu and Sundara [7]. If bilingualism itself confers an advantage due to better statistical learning abilities, then Spanish-English learning bilinguals should show accelerated word segmentation abilities when tested on Spanish trochees (this study) and Spanish iambs (previously tested in Mateu and Sundara [7]) as well, analogous to their accelerated ability to segment English iambs. Recall that the transitional probability cues are comparable for all three sets of stimuli. However, bilingual 8-month-olds failed to segment Spanish iambs, just like monolingual Spanish infants, showing no evidence for acceleration in Mateu and Sundara [7]. In the present study, again, there were no differences in the performance of the two groups: bilingual Spanish-English learning infants as well as monolingual Spanish-learning infants demonstrated segmentation of Spanish trochees in Block 3.

An addictiveness-based account also fails to account for our findings. We have argued previously in [7] that the determining factor in predicting accelerated segmentation of English iambs by bilingual infants was addictiveness (i.e., frequency effects). In English, only 10% of words start with a stressed syllable, whereas in Spanish the proportion is greater, 40% [70]. Thus, because of their Spanish experience, bilingual infants hear many more iambs than monolingual infants, which positively influences their ability to segment those from fluent speech in English. Consistent with the additiveness account, we expected to see accelerated segmentation of Spanish trochees by bilingual infants in the current study because in English, 90% of content words start with a stressed syllable [1], compared to 60% in Spanish [72]. However, this was not the case. That is, the greater frequency of a linguistic feature in one language does not inevitably lead to acceleration in the other language.

Instead, we propose that the findings of acceleration in segmenting English iambs, but not Spanish trochees, are owing to the fact that iambs are highly infrequent in English (10%), whereas trochees are not as rare in Spanish (40%). We know that infrequent patterns are underlearned cross-linguistically, early in acquisition, e.g., [79,80,81,82]. In artificial language experiments in the lab as well, learners are often biased to “sharpen” statistical distributions in their input, pushing them to prefer probabilities closer to 0 or 1, thus producing near-categorical learning outcomes [83,84,85]. This is especially true for children [86,87]. That is, young learners often fail to acquire infrequent patterns early in acquisition. This may be because they fail to encode infrequent patterns due to memory limitations [84]. Or because they regularize variability in the input [83,86] (see also [88,89]). Or because they filter infrequent patterns out as noise [82,90].

Whatever the cause, we see its effects during the development of word segmentation abilities in monolingual English-learning infants. English-learning 8-month-olds associate stressed syllables with word onsets, consistent with English input, where 90% of the words start with a stressed syllable. At the same age, they temporarily fail to segment English iambs like ‘guitar’ and ‘surprise’ [4], which constitute only 10% of English input [2]. 

No such underlearning is likely to be observed for the segmentation of Spanish trochees because trochees constitute 60% of Spanish input. Therefore, only bilingual Spanish-English learning infants’ segmentation of English iambs (not Spanish trochees) benefits from the additive effect of input from the other language. 

Extrapolating from these results to bilingual acquisition, we conclude that acceleration will not necessarily be observed when one language simply has more evidence for a pattern than the other. Instead, we expect it to emerge when one language has minimal evidence for a pattern and the other has substantially more instances of this pattern. Because of the additiveness of evidence from one language for the infrequent pattern, learners do not regularize, fail to encode, or filter out the infrequent pattern in the other.

In this paper, we focused our attention on identifying conditions for accelerated development in word segmentation in Spanish- and English-learning bilingual infants. We propose that it is only the learning of infrequent patterns in one language that may benefit from increased experience with the same pattern in another language. In addition to making falsifiable empirical predictions, our proposal restricts the cases—whether phonological or morpho-syntactic—where development may be accelerated. This is necessary because accelerated development in bilinguals is not common, so we need to be able to pinpoint fruitful areas of research if we want a window into language representation in bilingual learners. Another strength of this proposal is that it relies on bilingual infants using the same core mechanisms as monolingual infants in order to learn both their native languages. What we did not do in this paper was investigate the interaction between the various accounts proposed to account for acceleration, although we do state in the introduction that the four proposed accounts are not mutually exclusive. To fully understand cross-linguistic interaction in developing bilinguals, future research exploring the acquisition of linguistic features where these proposals intersect is necessary.

## 5. Conclusions

In sum, when bilingual learning children exhibit an accelerated rate of linguistic development compared to monolingual children, we can gain a better understanding of language representation in bilinguals if we are able to rule out explanations originating in imbalanced input or bilinguals’ reliance on domain-general mechanisms. In two experiments, we showed that, just like their monolingual Spanish-learning peers, bilingual Spanish-English-learning 8-month-olds successfully segment Spanish trochees and not just the stressed syllable. These results cannot be explained by language dominance, accounts appealing to perceptual salience, or bilingual infants reliance on domain-general abilities. They also cannot be attributed merely to increased exposure to a pattern in one language compared to another. That is, increased exposure to a linguistic feature does not automatically result in accelerated development. We argue that only the acquisition of infrequent patterns in one language can be accelerated by the additive effects of exposure to the other language. As a result, bilingual Spanish-English-learning infants segment infrequent English iambs, but not the more frequent Spanish trochees, earlier than their monolingual peers.

## Data Availability

Deidentified data are available from the OSF page for this project: https://osf.io/rncmq/, accessed on 31 October 2023.

## Appendix A

**Table A1:** Spanish passages with CVC.CV trochaic target words.

Gancho passage *Ese **gancho** le rasgó la camiseta. Se necesita un **gancho** para pescar. Mi madre colgó el abrigo en el **gancho**. El ladrón le hirió con el **gancho**. Hay un **gancho** detrás de la puerta. El **gancho** sobresalía de la pared.*
Salsa passage *Esa **salsa** era muy picante. Me encanta la **salsa** de mi abuela. Se ensució la ropa con una **salsa**. No es tan difícil escoger una **salsa**. Busca la **salsa** para la pasta. La **salsa** bechamel es mi favorita.*
Gesto passage *Un **gesto** bonito siempre gusta. No me gustó el **gesto** que me hizo. Le dije que viniera con un **gesto**. Cuando lo echó le hizo este **gesto**. Juan le hizo un **gesto** de aprobación. El **gesto** de Mona Lisa es un misterio.*
Venda passage *La **venda** le tapaba la rodilla. Quítate ya la **venda** de los ojos. Se cubrió la herida con esa **venda**. La enfermera le puso una **venda**. Llevaba una **venda** en la mano. La **venda** le apretaba demasiado.*
